# Complete Blood Count Parameters and Hemogram-Derived Inflammatory Indices in Adolescent Pregnancy: A Retrospective Comparative Cohort Study with Exploratory Machine-Learning Analyses

**DOI:** 10.3390/jcm15176721

**Published:** 2026-08-29

**Authors:** Meryem Bekmezci, Ramazan Bülbül, Şerife Özlem Genç, Hüseyin Erdal

**Affiliations:** 1 Department of Obstetrics and Gynecology, Faculty of Medicine, Aksaray University, 68100 Aksaray, Turkey; dr.ramazan_bulbul@hotmail.com; 2 Department of Obstetrics and Gynecology, Faculty of Medicine, Sivas Cumhuriyet University, 58140 Sivas, Turkey; serifeozlemgenc@cumhuriyet.edu.tr; 3 Department of Medical Genetics, Faculty of Medicine, Aksaray University, 68100 Aksaray, Turkey; herdalyfa@gmail.com

**Keywords:** adolescent pregnancy, machine-learning, systemic inflammation, hemogram-derived inflammatory indices, neonatal outcomes, complete blood count

## Abstract

**Background/Objectives**: Adolescent pregnancy is associated with an increased risk of adverse perinatal outcomes; however, its hematological characteristics remain incompletely understood. The aim of this study was to compare antepartum and postpartum complete blood count (CBC) parameters together with hemogram-derived inflammatory indices in adolescent and adult pregnancies and to investigate their associations with maternal and neonatal outcomes. **Methods**: This retrospective comparative cohort study included 428 singleton pregnancies comprising 205 adolescent (14–17 years) and 223 adults (18–42 years). Antepartum blood samples were obtained at admission for delivery, whereas postpartum samples were collected approximately six hours after delivery according to the institutional practice. CBC parameters (including neutrophil, lymphocyte, monocyte, and platelet counts) were recorded, and hemogram-derived inflammatory indices, including neutrophil-to-lymphocyte ratio (NLR), Platelet-to-lymphocyte ratio (PLR), systemic immune-inflammation index (SII), systemic inflammation response index (SIRI), aggregate index of systemic inflammation (AISI), and systemic inflammation-monocyte index (SIMI), were subsequently calculated. Comparisons were adjusted for gestational age at sampling and parity using a prespecified non-redundant marker set, and multiple testing was controlled using the Benjamini-Hochberg false discovery rate procedure. Multivariable logistic regression and machine-learning analyses were performed to evaluate associations with adverse maternal and neonatal outcomes. **Results:** Adolescent pregnancies demonstrated significantly higher antepartum neutrophil and monocyte counts than adult pregnancies (q < 0.05), and these differences remained significant after adjustment for gestational age at sampling and parity (both q < 0.01), whereas differences in hemoglobin and platelet counts were no longer significant (both q = 0.138). Low birth weight (10.2% vs. 4.5%), neonatal intensive care unit admission (15.1% vs. 8.5%), composite adverse outcome (22.9% vs. 15.2%), and birth weight below the 10th percentile (14.7% vs. 8.0%) were numerically more frequent among adolescents but were not statistically significant after false discovery rate correction (all q > 0.05). Across 30 prespecified marker–outcome associations, none remained significant after false discovery rate correction. Antepartum neutrophil count was not independently associated with low birth weight after multivariable adjustment (OR 1.233, 95% CI 0.858–1.737; *p* = 0.248, q = 0.413). Exploratory machine-learning models demonstrated only modest discrimination, with the highest cross-validated AUC of approximately 0.60, and did not outperform conventional regression models. **Conclusions**: Adolescent pregnancy was associated with higher neutrophil and monocyte counts during both the antepartum and postpartum periods, reflecting modest differences in hematological profile rather than an independently predictive inflammatory profile. These hematological differences were not independently associated with adverse maternal or neonatal outcomes after adjustment for confounding factors and correction for multiple testing. Hemogram-derived inflammatory indices should therefore be interpreted as mathematical transformations of routine CBC parameters rather than biologically independent biomarkers. Because blood samples were obtained at admission for delivery, these findings characterize the peripartum inflammatory profile and should not be interpreted as evidence of early pregnancy risk prediction.

## 1. Introduction

Adolescent pregnancy remains a global public health problem with significant consequences for maternal and neonatal health. Literature reports that low birth weight, preterm birth, need for neonatal intensive care, and adverse perinatal outcomes are more common in adolescent mothers compared to adult pregnancies [1,2,3,4,5]. Large-sample meta-analyses and multicenter cohort studies have shown that neonatal risks are more pronounced, particularly in pregnancies under the age of 17 [2,3]. Therefore, adolescent pregnancy can be considered not only a social and obstetric problem but also a physiological state associated with unique hematological and immunological adaptations that warrant further investigation.

The causes of adverse outcomes in adolescent pregnancies are not fully understood. While factors such as low socioeconomic status, inadequate antenatal care, nutritional deficiencies, and education level play a significant role, these variables are insufficient to explain the entire increase in risk observed in adolescent pregnancies [3,4]. In recent years, the concept of biological immaturity has come to the forefront; it has been proposed that ongoing somatic growth, hormonal maturation, and immunological adaptation processes during adolescence may affect pregnancy physiology differently than in adult pregnant women [6,7].

Pregnancy is a complex and dynamic process requiring the maternal immune system to develop tolerance to the fetus while simultaneously maintaining defense against infections [8,9,10]. Balanced regulation of the inflammatory response is necessary during implantation, placentation, fetal growth, birth, and postpartum tissue repair [11]. Disruption of this balance has been associated with adverse outcomes such as preterm birth, fetal growth restriction, hypertensive pregnancy disorders, and neonatal morbidity [12,13]. Hemogram-derived inflammatory indices, including the neutrophil-to-lymphocyte ratio (NLR), platelet-lymphocyte ratio (PLR), systemic immune-inflammatory index (SII), systemic inflammatory response index (SIRI), and aggregate systemic inflammation index (AISI), are calculated from routine complete blood count (CBC) parameters and have been widely used in obstetric research [14,15,16]. Because these indices are derived from routine CBC parameters, they provide an inexpensive and readily available summary of the underlying response in clinical practice.

Although there are numerous studies in the current literature examining the perinatal outcomes of adolescent pregnancies, studies evaluating the antepartum and postpartum inflammatory profile together in adolescent pregnancies are limited [1,6,7]. Furthermore, whether the inflammatory response in the postpartum period differs between adolescent and adult pregnant women has not been sufficiently investigated. Therefore, this study aimed to compare antepartum and postpartum inflammation indices derived from CBC in adolescent and adult pregnancies and to evaluate the relationship of these markers with maternal-neonatal outcomes. This study addresses an important knowledge gap by integrating conventional statistical analyses with machine-learning approaches to determine whether hemogram-derived inflammatory indices may provide clinically useful complementary measures of inflammatory changes rather than representing biologically independent biomarkers. We hypothesized that adolescent pregnancies would demonstrate differences in CBC parameters and corresponding hemogram-derived inflammatory indices compared with adult pregnancies during both the antepartum and postpartum periods.

## 2. Materials and Methods

### 2.1. Study Design and Population

**Figure 1:**
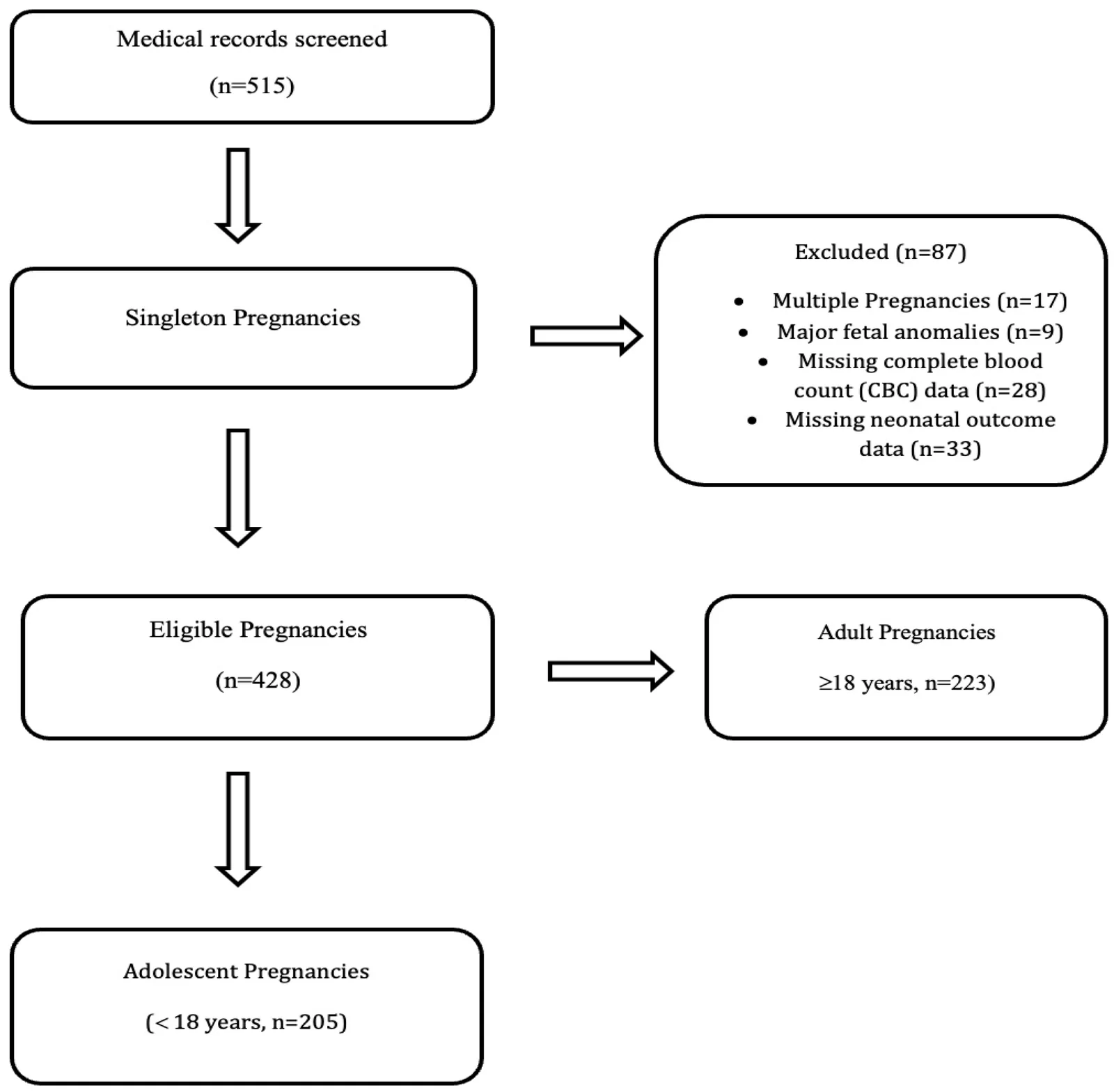
Flowchart of participant selection and study design.

This retrospective comparative cohort study was conducted at the Department of Obstetrics and Gynecology of a tertiary referral center. Medical records of pregnant women who delivered at the institution during the study period were reviewed. The study population consisted of 428 singleton pregnancies, including 205 adolescent pregnancies (14–17 years) and 223 adult pregnancies (18–42 years). Group allocation was based exclusively on maternal chronological age at delivery. Women with incomplete laboratory records, multiple pregnancies, major fetal anomalies, or missing outcome data were excluded from the analysis. The study was conducted in accordance with the principles of the Declaration of Helsinki and was approved by the Aksaray University Health Sciences Scientific Researches Ethics Committee (approval number: SAGETIK 2026/97 and Date: 30 April 2026). The participant selection process and study flow are summarized in Figure 1.

### 2.2. Data Collection

Demographic, obstetric, laboratory, and neonatal data were retrieved from the hospital electronic medical record system. Maternal age, parity, mode of delivery, gestational age at delivery, birth weight, newborn gender, neonatal intensive care unit (NICU) admission, and APGAR scores were recorded. Antepartum CBC measurements were routinely obtained at hospital admission before delivery. Postpartum CBC measurements obtained after delivery were also collected. Hemoglobin, neutrophil count, lymphocyte count, monocyte count, and platelet count were recorded for both time points. According to the institutional postpartum care protocol, postpartum CBC analysis was routinely performed approximately 6 h after delivery in all participants, irrespective of the mode of delivery.

### 2.3. Calculation of Hemogram-Derived Inflammatory Indices

The following inflammation indices were calculated using routinely available CBC parameters:

- Neutrophil-to-lymphocyte ratio (NLR) = Neutrophil count/Lymphocyte count
- Platelet-to-lymphocyte ratio (PLR) = Platelet count/Lymphocyte count
- Systemic immune-inflammation index (SII) = Neutrophil × Platelet/Lymphocyte
- Systemic inflammation response index (SIRI) = Neutrophil × Monocyte/Lymphocyte
- Systemic inflammation-monocyte index (SIMI) = Monocyte × Platelet /Lymphocyte
- Aggregate index of systemic inflammation (AISI) = Neutrophil × Monocyte × Platelet/Lymphocyte

All indices were calculated separately for antepartum and postpartum measurements.

### 2.4. Study Outcomes

The primary outcomes of interest were low birth weight (LBW), defined as a birth weight below 2500 g, and preterm birth, defined as a delivery before 37 completed weeks of gestation. Secondary outcomes included NICU admission, cesarean delivery, postpartum hemoglobin decline, and a composite adverse outcome defined as the occurrence of at least one of the following: LBW, preterm birth, or NICU admission.

### 2.5. Statistical Analysis

All statistical analyses were performed using Python version 3.10 (Python Software Foundation, Wilmington, DE, USA). Continuous variables were assessed for normality using the Shapiro-Wilk test. Normally distributed variables were compared using Student’s *t*-test or Welch’s *t*-test, whereas non-normally distributed variables were compared using the Mann-Whitney U test. Categorical variables were analyzed using the chi-square test or Fisher’s exact test, as appropriate. Changes between antepartum and postpartum measurements were evaluated using paired statistical tests according to data distribution. Correlation analyses were performed using Pearson’s or Spearman’s correlation coefficients. To account for multiple comparisons, *p*-values were adjusted using the Benjamini-Hochberg false discovery rate (FDR) procedure. Multivariable logistic regression analyses were performed to identify variables independently associated with adverse outcomes. To avoid mathematical redundancy, confirmatory multivariable models were prespecified using a non-redundant marker set informed by biological interpretation and multicollinearity assessment rather than data-driven variable selection.

### 2.6. Machine-Learning Analysis

In addition to conventional statistical analyses, machine-learning models were implemented as exploratory analyses to evaluate the predictive performance of maternal demographic characteristics, complete blood count parameters, and hemogram-derived inflammatory indices for adverse neonatal outcomes. Penalized logistic regression, Random Forest (RF) and Extreme Gradient Boosting (XGBoost) algorithms were trained using stratified five-fold cross-validation. To address class imbalance, synthetic minority oversampling (SMOTE) was applied exclusively to training datasets. Model performance was evaluated using the area under the receiver operating characteristics curve (AUC), precision-recall metrics, calibration analyses, and Brier scores. Feature importance was explored using Shapley Additive exPlanations (SHAP) to improve model interpretability. A two-sided *p*-value < 0.05 was considered statistically significant. Given the limited number of outcome events, the machine-learning analyses were considered exploratory and hypothesis-generating rather than confirmatory.

## 3. Results

### 3.1. Baseline Characteristics

A total of 428 singleton pregnancies were included in the final analysis, comprising 205 adolescents and 223 adults. The adolescent group included participants aged 14–17 years, whereas the adult group ranged from 18 to 42 years. As expected based on the predefined study groups, maternal age and parity differed significantly between adolescents and adults (both q < 0.01). Median birth weight was also lower in the adolescent group (3135 g [2770–3470] vs. 3240 g [2960–3532], q = 0.013). Among antepartum hematological parameters, adolescents had lower hemoglobin concentrations but higher neutrophil, monocyte, platelet, and NLR values than adults, whereas lymphocyte counts and PLR were comparable between groups. Postpartum, adolescents likewise had lower hemoglobin levels and higher neutrophil, monocyte, platelet, and NLR values, while lymphocyte counts and PLR remained similar. Baseline demographic, hematological, and obstetric characteristics are summarized in Table 1.

### 3.2. Adjustment for Gestational Age and Parity

Because adolescents delivered at a slightly earlier gestational age and had lower parity than adults, group differences in antepartum hematological markers were re-evaluated after adjustment for these variables. Following adjustment, higher neutrophil and monocyte counts and a higher neutrophil-to-lymphocyte ratio (NLR) remained independently associated with adolescent pregnancy (all q ≤ 0.003). In contrast, the between-group differences in hemoglobin concentration and platelet count were attenuated and were no longer statistically significant after adjustment (both q = 0.138). No significant difference was observed for lymphocyte count or platelet-to-lymphocyte ratio (PLR). These findings indicate that the principal hematological differences between adolescents and adults were confined to neutrophil- and monocyte-related inflammatory markers rather than to hemoglobin or platelet parameters (Table 2).

### 3.3. Maternal and Neonatal Outcomes

Maternal and neonatal outcomes are summarized in Table 3. Although adolescents showed numerically higher rates of low birth weight, neonatal intensive care unit admission, composite adverse outcome, and birth weight below the 10th percentile, none of these differences remained statistically significant after false discovery rate correction. Likewise, no significant between-group differences were observed for preterm birth, mode of delivery, macrosomia, very low birth weight, or 5-min Apgar score below 7 after adjustment for multiple comparisons (Table 3).

### 3.4. Multicollinearity Assessment of Inflammation-Derived Indices

**Figure 2:**
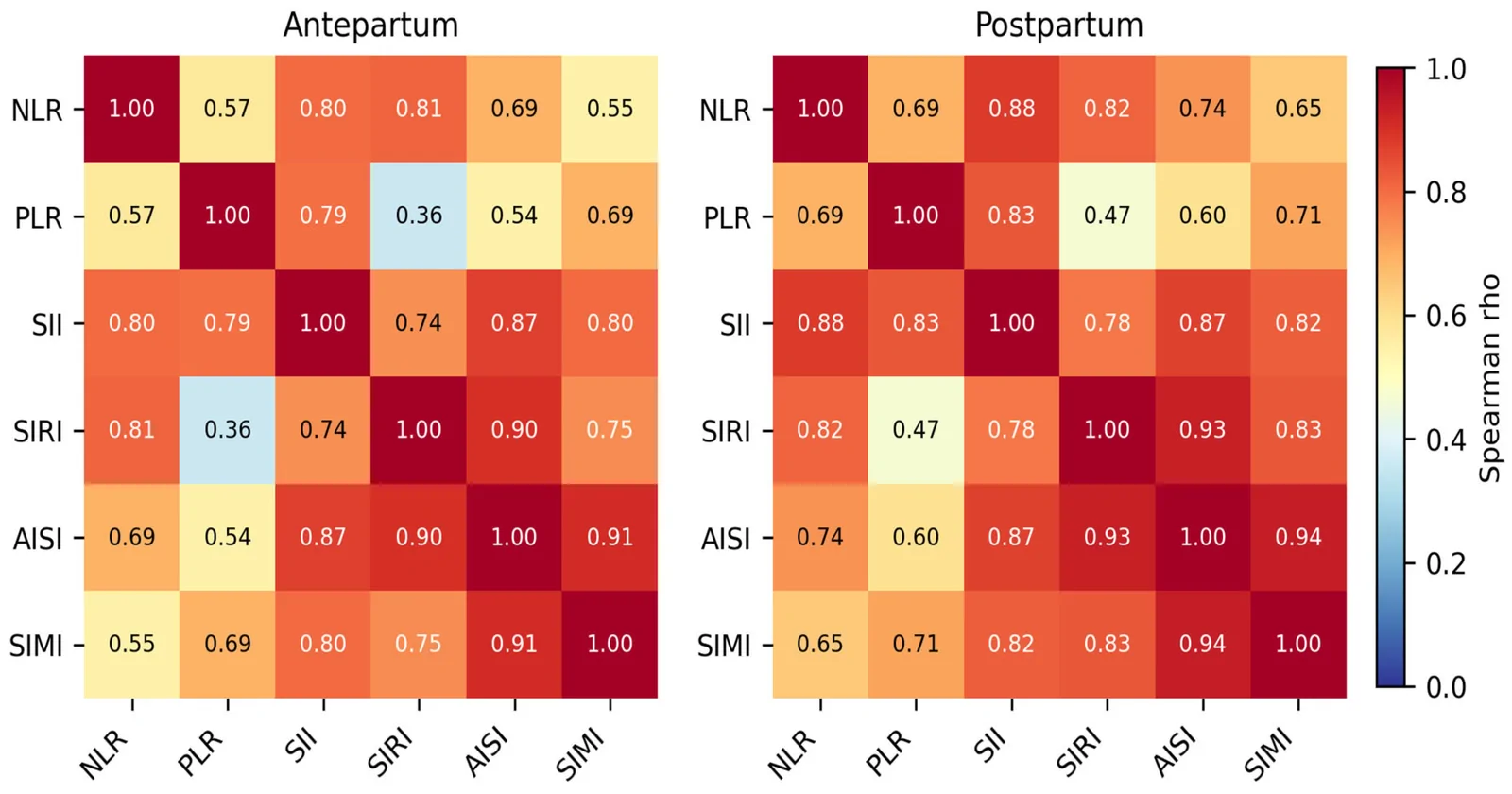
Pairwise Spearman correlation among the six hemogram derived indices.

Because several inflammation-derived indices are calculated from the same CBC components, the relationships among these variables were evaluated before multivariable modelling. Pairwise correlation analysis demonstrated strong correlations between several derived indices, indicating substantial shared information among these markers (Figure 2). Consistent with these findings, multicollinearity assessment showed that models including all derived indices simultaneously exhibited severe collinearity, whereas models restricted to complete blood count variables alone demonstrated acceptable variance inflation factors. These findings supported modelling based on the prespecified non-redundant marker set (Table 4).

### 3.5. Multivariable Logistic Regression Analyses

Multivariable logistic regression analyses were performed using prespecified models in which one hematological marker was entered at a time together with the relevant clinical covariates. Collinearity diagnostics supported this modelling strategy, and all candidate markers were therefore evaluated individually. Across the 30 prespecified marker–outcome associations, only one association reached nominal statistical significance, whereas none remained significant after false discovery rate correction. The smallest adjusted q value was 0.339, indicating no statistically significant independent association between any antepartum hematological marker and the evaluated maternal or neonatal outcomes. For low birth weight, antepartum neutrophil count was not independently associated with the outcome after adjustment (OR 1.233, 95% CI 0.858–1.737, *p* = 0.248, q = 0.413). Likewise, none of the remaining hematological markers demonstrated statistically significant independent associations with any evaluated maternal or neonatal outcome after false discovery rate correction (Table 5).

### 3.6. Exploratory Machine-Learning Analyses

Exploratory machine learning analyses were performed using penalized logistic regression, random forest, and XGBoost models. Overall, discrimination was modest across all outcomes. Random forest consistently achieved the highest cross-validated AUC; however, calibration remained suboptimal and predictive performance was insufficient for clinical application. These exploratory findings were consistent with the confirmatory regression analyses, indicating limited incremental predictive value of antepartum hematological markers (Table S1). Representative ROC curves and calibration plots are provided in Figure S1, while null distributions obtained by permuting the outcome labels are presented in Figure S2 (Supplementary Materials).

## 4. Discussion

This retrospective cohort study compared antepartum hematological parameters and complete blood count-derived inflammatory indices between adolescent and adult pregnancies and evaluated their associations with maternal and neonatal outcomes. Four principal findings emerged. First, after adjustment for gestational age and parity, adolescent pregnancies remained characterized by higher antepartum neutrophil and monocyte counts and a higher neutrophil-to-lymphocyte ratio than adult pregnancies, whereas differences in hemoglobin and platelet counts were no longer statistically significant. Second, despite these hematological differences, none of the evaluated hematological markers demonstrated an independent association with adverse maternal or neonatal outcomes after multivariable adjustment and false discovery rate correction. Third, multicollinearity analyses confirmed that several derived inflammatory indices contained substantial redundant information because they were mathematically derived from the same complete blood count components, supporting the use of a prespecified non-redundant marker set in confirmatory analyses. Finally, exploratory machine-learning analyses showed only modest predictive performance, consistent with the limited prognostic value observed in the conventional regression analyses.

Previous studies have consistently demonstrated that adolescent pregnancy is associated with an increased risk of adverse maternal and neonatal outcomes, particularly low birth weight, preterm birth, and neonatal morbidity compared with adult pregnancy [1,4,5]. Large multicenter studies have further shown that these risks increase with decreasing maternal age, suggesting that both biological immaturity and social determinants may contribute to adverse pregnancy outcomes [2,3,6,7]. However, in the present cohort, although adolescents showed numerically higher rates of low birth weight, neonatal intensive care unit admission, composite adverse outcome, and birth weight below the 10th percentile, none of these differences remained statistically significant after false discovery rate correction. This discrepancy does not necessarily contradict previous reports but rather indicates that, after controlling for multiple testing, the observed between-group differences were not sufficiently robust to support independent statistical significance in our cohort. The relatively low frequency of several adverse outcomes together with the conservative nature of false discovery rate adjustment may also have reduced the statistical power to detect modest between-group differences. Nevertheless, the direction of the observed associations remained consistent with previous systematic reviews and large population-based studies evaluating adolescent pregnancy [1,3,4]. The biological mechanisms underlying adverse outcomes in adolescent pregnancy are likely multifactorial and cannot be explained solely by socioeconomic or environmental factors. Ongoing somatic growth, endocrine maturation, and age-related adaptations of the maternal immune system may contribute to differences in hematological and inflammatory responses during pregnancy [6,7,8,9,10]. Consistent with this concept, adolescents in our cohort exhibited higher antepartum neutrophil and monocyte counts as well as a higher neutrophil-to-lymphocyte ratio after adjustment for gestational age and parity. These findings indicate modest group-level hematological differences that may reflect physiological adaptations associated with adolescent pregnancy rather than overt pathological inflammation. However, because none of these hematological markers remained independently associated with adverse maternal or neonatal outcomes after multivariable adjustment and false discovery rate correction, these differences should be interpreted as reflecting group-level hematological variation rather than clinically useful prognostic biomarkers [8,9,10,12,13,14,15,16]. Pregnancy is characterized by dynamic and tightly regulated immune adaptations that vary across gestation and are essential for successful implantation, fetal tolerance, parturition, and postpartum tissue repair [8,9,10,11]. Disruption of this physiological immune balance has been implicated in several obstetric complications, including preterm birth and hypertensive disorders of pregnancy [10,11]. Consequently, complete blood count-derived inflammatory indices such as the neutrophil-to-lymphocyte ratio, systemic immune-inflammation index, and related composite markers have gained increasing attention as inexpensive and readily available surrogate markers of systemic inflammation in obstetric research [12,13,14]. However, the clinical utility of these indices remains controversial, with previous studies reporting inconsistent and often modest predictive performance across different pregnancy complications [14,15,16].

Our findings are consistent with this growing body of evidence, indicating that although several inflammatory markers differed between adolescent and adult pregnancies at the group level, they did not provide meaningful independent prognostic information for adverse maternal or neonatal outcomes after adjustment for confounding factors and correction for multiple testing [12,13,14,15,16]. The observed differences in hematological parameters should also be interpreted in the context of the mathematical structure of complete blood count-derived inflammatory indices. Most composite indices share common cellular components, particularly neutrophils, lymphocytes, and monocytes, resulting in substantial overlap in the biological information they convey. Consistent with this concept, our multicollinearity analysis demonstrated considerable redundancy among several derived inflammatory indices, supporting the use of a prespecified non-redundant marker set for confirmatory analyses. Collectively, these findings suggest that statistically significant differences across multiple derived indices do not necessarily represent independent biological processes but may largely reflect variation in the underlying complete blood count parameters from which they are calculated. Therefore, caution is warranted when interpreting multiple significant inflammation indices as evidence of distinct inflammatory pathways.

The exploratory machine-learning analyses further supported the findings of the conventional statistical models. Although Random Forest consistently achieved the highest cross-validated discriminatory performance, the overall predictive accuracy of all evaluated models remained modest and insufficient for clinical application. These findings reinforce the conclusion that the evaluated hematological markers provide limited incremental prognostic information beyond conventional statistical approaches. The concordance between multivariable regression and exploratory machine-learning analyses strengthens the robustness of our findings and suggests that the limited predictive performance reflects the characteristics of the evaluated biomarkers rather than the analytical method applied. Accordingly, complete blood count-derived inflammatory indices may be more appropriately regarded as complementary indicators of underlying hematological variation than as reliable standalone tools for individualized risk prediction in adolescent pregnancies. From a clinical perspective, the present findings suggest that modest elevations in complete blood count-derived inflammatory indices observed in adolescent pregnancies should be interpreted with caution. Although adolescents exhibited a distinct hematological profile compared with adult pregnant women, these differences did not translate into independent prediction of adverse maternal or neonatal outcomes after multivariable adjustment and correction for multiple testing. Therefore, isolated elevations in these indices should not automatically be considered evidence of pathological inflammation but rather interpreted within the broader clinical context, given the physiological immune adaptations that normally occur during pregnancy. Future prospective multicenter studies incorporating hematological, immunological, hormonal, metabolic, nutritional, and socioeconomic variables are warranted to better characterize the biological mechanisms underlying adolescent pregnancy and to determine whether complete blood count-derived inflammatory markers provide clinically meaningful prognostic information beyond conventional risk factors. This study has several limitations. First, its retrospective single-center design precludes causal inference and may limit the generalizability of the findings. Second, although the regression analyses were adjusted for available covariates, several potentially important confounding factors, including body mass index, smoking status, nutritional status, medication use, socioeconomic characteristics, and detailed obstetric comorbidities, were not consistently available because of the retrospective design and therefore could not be incorporated into the final models. Consequently, residual confounding cannot be completely excluded. Third, inflammatory cytokines, C-reactive protein, interleukins, and other molecular biomarkers were not available; therefore, the observed hematological differences could not be validated using complementary immunological markers. Fourth, although antepartum blood samples were routinely obtained before delivery according to institutional clinical practice, the retrospective design did not allow complete standardization of all preanalytical and clinical factors that might influence hematological parameters. Finally, although multiple comparison correction reduced the risk of false-positive findings, it may also have decreased statistical power to detect modest associations, particularly for relatively infrequent maternal and neonatal outcomes. In addition, the exploratory machine-learning models were not externally validated in an independent cohort, and their findings should therefore be interpreted as hypothesis-generating rather than confirmatory.

## 5. Conclusions

In conclusion, adolescent pregnancies were characterized by modest differences in antepartum hematological parameters, particularly higher neutrophil and monocyte counts and a higher neutrophil-to-lymphocyte ratio after adjustment for gestational age and parity. However, these hematological differences did not translate into independent prediction of adverse maternal or neonatal outcomes after multivariable adjustment and false discovery rate correction. Multicollinearity and exploratory machine-learning analyses further supported the limited incremental prognostic value of complete blood count-derived inflammatory indices. These findings suggest that such indices should be regarded as complementary indicators of underlying hematological variation rather than reliable standalone biomarkers for individualized risk prediction in adolescent pregnancies. Future prospective multicenter studies integrating hematological, immunological, and clinical risk factors are needed to better define the biological mechanisms underlying adolescent pregnancy and to identify clinically useful prognostic biomarkers. Prospective studies with standardized sampling protocols and external validation are warranted to further clarify the biological and clinical relevance of these findings.

## Figures and Tables

**Table 1:** Baseline demographic, obstetric, and hematological characteristics of adolescent and adult pregnancies.

Variable	Adolescent	Adult	Test	*p*	q, 38 Test Family	q, 30 Test Family	Role in the Analysis
Maternal age (years)	17.00 [16.00 to 17.00]	27.00 [24.00 to 33.00]	Mann Whitney U	<0.001	<0.001	<0.001	design variable
Parity	1.00 [1.00 to 2.00]	3.00 [2.00 to 4.00]	Mann Whitney U	<0.001	<0.001	<0.001	design variable
Gestational age at delivery (weeks)	39.00 [38.00 to 40.00]	39.30 [38.30 to 40.40]	Mann Whitney U	<0.001	<0.001	<0.001	design variable
Birth weight (g)	3135.00 [2770.00 to 3470.00]	3240.00 [2960.00 to 3532.50]	Mann Whitney U	0.008	0.011	0.013	outcome or derived outcome
Antepartum hemoglobin (g/dL)	12.00 [11.00 to 13.10]	12.40 [11.40 to 13.20]	Mann Whitney U	0.035	0.041	0.043	primary marker
Antepartum neutrophil (×10^9^/L)	9.17 [7.61 to 11.35]	7.42 [6.01 to 9.44]	Mann Whitney U	<0.001	<0.001	<0.001	primary marker
Antepartum lymphocyte (×10^9^/L)	1.98 [1.62 to 2.37]	1.88 [1.52 to 2.33]	Mann Whitney U	0.346	0.359	0.364	primary marker
Antepartum monocyte (×10^9^/L)	0.61 [0.50 to 0.74]	0.51 [0.43 to 0.64]	Mann Whitney U	<0.001	<0.001	<0.001	primary marker
Antepartum platelet (×10^9^/L)	242.00 [207.00 to 292.00]	229.00 [183.50 to 269.00]	Mann Whitney U	0.004	0.005	0.006	primary marker
Antepartum NLR	4.35 [3.46 to 6.48]	3.91 [3.12 to 4.91]	Mann Whitney U	<0.001	<0.001	<0.001	secondary marker
Antepartum PLR	123.31 [93.02 to 164.89]	117.75 [94.03 to 151.24]	Mann Whitney U	0.191	0.214	0.225	secondary marker
Postpartum hemoglobin (g/dL)	10.71 ± 1.51	11.21 ± 1.37	Welch t	<0.001	<0.001	<0.001	primary marker
Postpartum neutrophil (×10^9^/L)	13.82 [11.29 to 17.02]	11.36 [9.03 to 14.11]	Mann Whitney U	<0.001	<0.001	<0.001	primary marker
Postpartum lymphocyte (×10^9^/L)	1.52 [1.18 to 1.90]	1.54 [1.13 to 1.87]	Mann Whitney U	0.668	0.668	0.668	primary marker
Postpartum monocyte (×10^9^/L)	0.83 [0.69 to 1.02]	0.69 [0.54 to 0.86]	Mann Whitney U	<0.001	<0.001	<0.001	primary marker
Postpartum platelet (×10^9^/L)	223.50 [186.50 to 266.25]	204.00 [169.00 to 250.00]	Mann Whitney U	0.009	0.012	0.013	primary marker
Postpartum NLR	9.06 [6.61 to 12.74]	7.59 [5.18 to 10.17]	Mann Whitney U	<0.001	<0.001	<0.001	secondary marker
Postpartum PLR	143.23 [107.65 to 192.83]	137.27 [104.07 to 180.18]	Mann Whitney U	0.206	0.222	0.229	secondary marker
Five minute Apgar score	10.00 [10.00 to 10.00]	10.00 [10.00 to 10.00]	Mann Whitney U	<0.001	<0.001	<0.001	outcome or derived outcome
Hemoglobin drop (g/dL)	1.20 [0.75 to 1.70]	1.00 [0.50 to 1.50]	Mann Whitney U	0.016	0.019	0.021	outcome or derived outcome
Antepartum SII	1046.03 [758.14 to 1770.65]	896.71 [669.18 to 1202.23]	Mann Whitney U	<0.001	<0.001	not applicable	descriptive only, not entered into any outcome model
Antepartum SIRI	2.82 [1.92 to 4.29]	2.04 [1.49 to 2.95]	Mann Whitney U	<0.001	<0.001	not applicable	descriptive only, not entered into any outcome model
Antepartum AISI	696.35 [434.63 to 1093.44]	464.67 [280.35 to 696.55]	Mann Whitney U	<0.001	<0.001	not applicable	descriptive only, not entered into any outcome model
Antepartum SIMI	77.54 [53.47 to 106.05]	61.70 [43.46 to 82.21]	Mann Whitney U	<0.001	<0.001	not applicable	descriptive only, not entered into any outcome model
Postpartum SII	1918.68 [1411.26 to 2945.15]	1555.00 [1028.00 to 2187.50]	Mann Whitney U	<0.001	<0.001	not applicable	descriptive only, not entered into any outcome model
Postpartum SIRI	7.51 [5.14 to 11.28]	5.27 [3.24 to 8.20]	Mann Whitney U	<0.001	<0.001	not applicable	descriptive only, not entered into any outcome model
Postpartum AISI	1576.71 [1123.21 to 2707.48]	1039.49 [589.82 to 1720.64]	Mann Whitney U	<0.001	<0.001	not applicable	descriptive only, not entered into any outcome model
Postpartum SIMI	117.83 [88.40 to 165.55]	94.53 [64.94 to 134.12]	Mann Whitney U	<0.001	<0.001	not applicable	descriptive only, not entered into any outcome model

Data are presented as median (interquartile range), mean ± standard deviation, or number (percentage), as appropriate. Continuous variables were compared using the Mann–Whitney U test or Welch’s *t*-test according to data distribution. *p* values were adjusted for multiple comparisons using the Benjamini–Hochberg false discovery rate (FDR) procedure. Derived inflammatory indices are presented for descriptive purposes only and were not included in multivariable models. Abbreviations: AISI, Aggregate Index of Systemic Inflammation; FDR, False Discovery Rate; NLR, Neutrophil-to-Lymphocyte Ratio; PLR, Platelet-to-Lymphocyte Ratio; SII, Systemic Immune-Inflammation Index; SIMI, Systemic Inflammation Monocyte Index; SIRI, Systemic Inflammation Response Index.

**Table 2:** Adjusted differences in antepartum hematological parameters between adolescent and adult pregnancies.

Marker	Crude Difference	Adjusted for Gestational Age	Adjusted for Gestational Age and Parity	*p*	q
Hemoglobin (g/dL)	−0.324	−0.252	−0.297	0.079	0.138
Neutrophil (×10^9^/L)	2.198	1.984	1.718	<0.001	<0.001
Lymphocyte (×10^9^/L)	0.019	0.003	−0.028	0.725	0.725
Monocyte (×10^9^/L)	0.106	0.092	0.084	0.001	0.003
Platelet (×10^9^/L)	20.665	20.033	12.693	0.099	0.138
NLR	1.518	1.353	1.277	<0.001	0.003
PLR	10.622	10.326	8.163	0.156	0.182

Adjusted estimates were obtained using multivariable linear regression models including gestational age at delivery and parity. Positive values indicate higher marker levels in adolescents compared with adults. *p* values were adjusted using the Benjamini–Hochberg FDR procedure. Abbreviations: NLR, Neutrophil-to-Lymphocyte Ratio; PLR, Platelet-to-Lymphocyte Ratio; FDR, False Discovery Rate.

**Table 3:** Maternal and neonatal outcomes in adolescent and adult pregnancies.

Variable	Adolescent	Adult	*p*	q, 38 Test Family	q, 30 Test Family
Newborn sex	Female = 91; Male = 114	Female = 108; Male = 115	0.459	0.529	0.551
Mode of delivery	Cesarean Section = 53; Vaginal Delivery = 152	Cesarean Section = 79; Vaginal Delivery = 144	0.042	0.061	0.069
NICU admission	31/205 (15.1%)	19/223 (8.5%)	0.048	0.068	0.076
Low birth weight	21/205 (10.2%)	10/223 (4.5%)	0.035	0.053	0.061
Preterm birth	21/205 (10.2%)	18/223 (8.1%)	0.541	0.600	0.614
Very low birth weight	0/205 (0.0%)	1/223 (0.4%)	1.000	1.000	1.000
Macrosomia	6/205 (2.9%)	10/223 (4.5%)	0.553	0.600	0.614
Five minute Apgar below 7	1/205 (0.5%)	1/221 (0.5%)	1.000	1.000	1.000
Composite adverse outcome	47/205 (22.9%)	34/223 (15.2%)	0.057	0.075	0.082
Birth weight below the tenth centile for gestational week and sex	27/184 (14.7%)	16/201 (8.0%)	0.054	0.073	0.081

Categorical variables are presented as number (percentage). Comparisons were performed using the chi-square test or Fisher’s exact test, as appropriate. *p* values were adjusted using the Benjamini–Hochberg FDR procedure. Abbreviations: FDR, False Discovery Rate; NICU, Neonatal Intensive Care Unit.

**Table 4:** Multicollinearity Assessment of Hematological Variables.

Predictor	All Candidates Including the Previously Excluded Indices	Cell Counts	Derived Indices
Antepartum AISI	78.689	NaN	NaN
Antepartum NLR	46.974	NaN	1.608
Antepartum PLR	17.903	NaN	1.587
Antepartum SII	64.636	NaN	NaN
Antepartum SIMI	37.012	NaN	NaN
Antepartum SIRI	77.715	NaN	NaN
Antepartum hemoglobin	1.140	1.115	NaN
Antepartum lymphocyte	6.612	1.362	NaN
Antepartum monocyte	15.098	1.780	NaN
Antepartum neutrophil	14.342	1.662	NaN
Antepartum platelet	10.359	1.266	NaN
Maternal age	1.804	1.795	1.721
Parity	1.698	1.692	1.684

Variance inflation factors (VIFs) were calculated under three model specifications. Values < 5 indicate acceptable multicollinearity, whereas substantially higher values indicate severe collinearity. The final multivariable models were based on the prespecified non-redundant marker set. Abbreviations: AISI; Aggregate Index of Systemic Inflammation; NLR, Neutrophil-to-Lymphocyte Ratio; PLR, Platelet-to-Lymphocyte Ratio; SII, Systemic Immune-Inflammation Index; SIMI, Systemic Inflammation Monocyte Index; SIRI, Systemic Inflammation Response Index; VIF, Variance Inflation Factor, NaN, Not a Number; indicates that the VIF was not calculated because the corresponding variable was not included in that model specification.

**Table 5:** Multivariable Logistic Regression Analysis of Hematological Markers and Adverse Pregnancy Outcomes.

Outcome	Marker	*n*	Events	Variables	Events per Variable	Odds Ratio per Standard Deviation	Lower	Upper	*p*	q
Low birth weight	Hemoglobin	428	31	5	6.200	1.364	0.886	2.156	0.161	0.339
Low birth weight	Neutrophil	428	31	5	6.200	1.233	0.858	1.737	0.248	0.413
Low birth weight	Lymphocyte	427	31	5	6.200	0.951	0.648	1.381	0.786	0.886
Low birth weight	Monocyte	428	31	5	6.200	0.900	0.578	1.294	0.597	0.814
Low birth weight	Platelet	428	31	5	6.200	1.343	0.909	1.936	0.134	0.339
Preterm birth	Hemoglobin	428	39	3	13.000	0.666	0.485	0.912	0.012	0.339
Preterm birth	Neutrophil	428	39	3	13.000	1.282	0.959	1.669	0.090	0.339
Preterm birth	Lymphocyte	427	39	3	13.000	1.257	0.923	1.677	0.141	0.339
Preterm birth	Monocyte	428	39	3	13.000	1.302	0.977	1.696	0.070	0.339
Preterm birth	Platelet	428	39	3	13.000	0.984	0.697	1.354	0.926	0.949
NICU admission	Hemoglobin	428	50	4	12.500	1.363	0.9995	1.887	0.050	0.339
NICU admission	Neutrophil	428	50	4	12.500	1.060	0.797	1.382	0.676	0.881
NICU admission	Lymphocyte	427	50	4	12.500	0.961	0.710	1.268	0.781	0.886
NICU admission	Monocyte	428	50	4	12.500	0.817	0.575	1.110	0.208	0.378
NICU admission	Platelet	428	50	4	12.500	1.010	0.740	1.350	0.949	0.949
Composite adverse	Hemoglobin	428	81	3	27.000	0.974	0.767	1.241	0.828	0.887
Composite adverse	Neutrophil	428	81	3	27.000	1.174	0.931	1.465	0.170	0.339
Composite adverse	Lymphocyte	427	81	3	27.000	1.248	0.989	1.571	0.062	0.339
Composite adverse	Monocyte	428	81	3	27.000	1.088	0.854	1.363	0.482	0.723
Composite adverse	Platelet	428	81	3	27.000	1.195	0.943	1.511	0.139	0.339
Cesarean section	Hemoglobin	428	132	4	33.000	0.861	0.698	1.060	0.158	0.339
Cesarean section	Neutrophil	428	132	4	33.000	0.829	0.645	1.041	0.108	0.339
Cesarean section	Lymphocyte	427	131	4	32.750	1.113	0.908	1.367	0.303	0.478
Cesarean section	Monocyte	428	132	4	33.000	0.940	0.749	1.161	0.571	0.814
Cesarean section	Platelet	428	132	4	33.000	1.028	0.832	1.265	0.797	0.886
Birth weight below the tenth centile	Hemoglobin	385	43	3	14.333	1.288	0.932	1.812	0.126	0.339
Birth weight below the tenth centile	Neutrophil	385	43	3	14.333	0.952	0.662	1.286	0.764	0.886
Birth weight below the tenth centile	Lymphocyte	384	43	3	14.333	0.809	0.566	1.124	0.214	0.378
Birth weight below the tenth centile	Monocyte	385	43	3	14.333	0.703	0.450	1.027	0.071	0.339
Birth weight below the tenth centile	Platelet	385	43	3	14.333	0.770	0.534	1.078	0.132	0.339

One hematological marker was entered into each multivariable logistic regression model together with the prespecified clinical covariates. Odds ratios are expressed per one standard deviation increase in each marker. *p* values were adjusted for multiple comparisons using the Benjamini–Hochberg FDR procedure across the 30 prespecified marker–outcome associations. Abbreviations: FDR, False Discovery Rate; OR, Odds Ratio.

## Data Availability

The datasets generated and/or analyzed during the current study are available from the corresponding author on reasonable request.

## Supplementary Materials

The following supporting information can be downloaded at: https://www.mdpi.com/article/10.3390/jcm15176721/s1. Table S1: Machine-Learning Performance without Oversampling; Figure S1: Receiver operating characteristic curves and calibration plots from out-of-fold predictions; Figure S2: Null distributions obtained by permuting the outcome labels, with the observed value marked.

## Abbreviations

The following abbreviations are used in this manuscript:

AISI	Aggregate Index of Systemic Inflammation
AUC	Area Under the Receiver Operating Characteristic Curve
BH	Benjamini–Hochberg
CBC	Complete Blood Count
CI	Confidence Interval
F1	F1 Score
LBW	Low Birth Weight
ML	Machine Learning
NICU	Neonatal Intensive Care Unit
NLR	Neutrophil-to-Lymphocyte Ratio
OR	Odds Ratio
PLR	Platelet-to-Lymphocyte Ratio
PR AUC	Precision–Recall Area Under the Curve
RF	Random Forest
ROC	Receiver Operating Characteristic
SHAP	SHapley Additive exPlanations
SIMI	Systemic Inflammation–Monocyte Index
SII	Systemic Immune-Inflammation Index
SIRI	Systemic Inflammation Response Index
SMOTE	Synthetic Minority Over-sampling Technique
VIF	Variance Inflation Factor
XGBoost	Extreme Gradient Boosting
APGAR	Appearance, Pulse, Grimace, Activity, Respiration
BMI	Body Mass Index
SD	Standard Deviation
SGA	Small for Gestational Age
VLBW	Very Low Birth Weight
