# Supplementary material for: Complete Blood Count Parameters and Hemogram-Derived Inflammatory Indices in Adolescent Pregnancy: A Retrospective Comparative Cohort Study with Exploratory Machine-Learning Analyses
Source: J Clin Med. 2026 Aug 29;15(17):6721. doi: 10.3390/jcm15176721 (PMC13566511; doi:10.3390/jcm15176721)
Supplement: Supplementary file 1 [file jcm-15-06721-s001.zip › jcm-4463050-supplementary.pdf]

Table S1. Machine-Learning Performance without oversampling

	Events	Model	Cross validated area	Standard deviation	Apparent area	Optimism	Precision recall area	Brier score	Calibration slope	Permutation p	q
Low birth weight	31	Penalised logistic	0.513	0.026	0.644	0.130	0.094	0.068	0.117	not applicable	not applicable
Low birth weight	31	Random forest	0.666	0.020	0.941	0.274	0.168	0.066	0.718	0.020	0.040
Low birth weight	31	XGBoost	0.604	0.029	0.986	0.382	0.102	0.073	0.213	not applicable	not applicable
Preterm birth	39	Penalised logistic	0.587	0.043	0.691	0.104	0.140	0.085	0.350	not applicable	not applicable
Preterm birth	39	Random forest	0.638	0.009	0.927	0.290	0.182	0.081	0.598	0.015	0.040
Preterm birth	39	XGBoost	0.536	0.016	0.957	0.420	0.122	0.089	0.078	not applicable	not applicable
NICU admission	50	Penalised logistic	0.481	0.039	0.588	0.107	0.129	0.104	0.083	not applicable	not applicable
NICU admission	50	Random forest	0.525	0.019	0.937	0.412	0.131	0.108	0.045	0.279	0.279
NICU admission	50	XGBoost	0.500	0.025	0.986	0.486	0.122	0.117	0.009	not applicable	not applicable
Composite adverse	81	Penalised logistic	0.469	0.007	0.587	0.117	0.189	0.157	-0.583	not applicable	not applicable
Composite adverse	81	Random forest	0.568	0.011	0.915	0.347	0.228	0.157	0.361	0.100	0.133
Composite adverse	81	XGBoost	0.530	0.026	0.983	0.453	0.204	0.175	0.065	not applicable	not applicable

Model performance was evaluated using repeated nested cross-validation. Random forest, penalized logistic regression, and XGBoost models were compared. Performance metrics are reported for exploratory analyses only and should not be interpreted as confirmatory evidence. Abbreviations: AUC, Area Under the Receiver Operating Characteristic Curve; PR-AUC, Area Under the Precision–Recall Curve; XGBoost, Extreme Gradient Boosting.

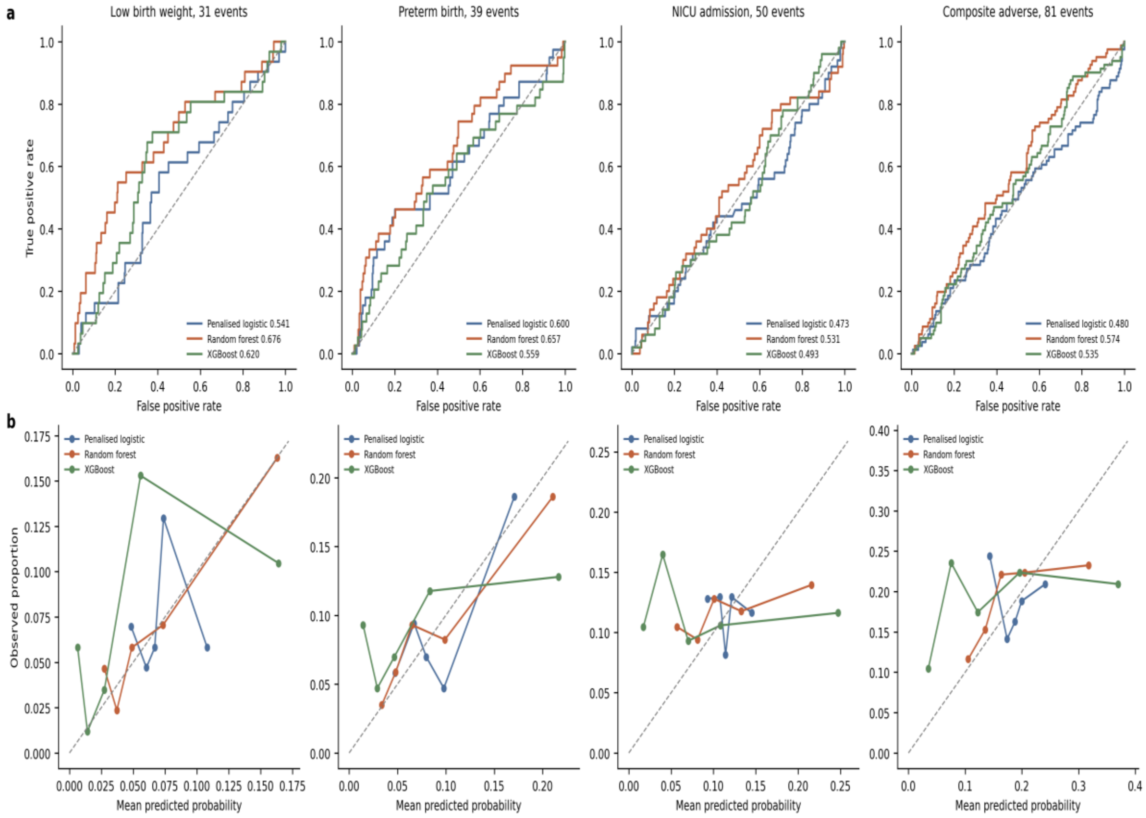

Figure S1. Receiver operating characteristic curves and calibration plots from out of fold predictions.

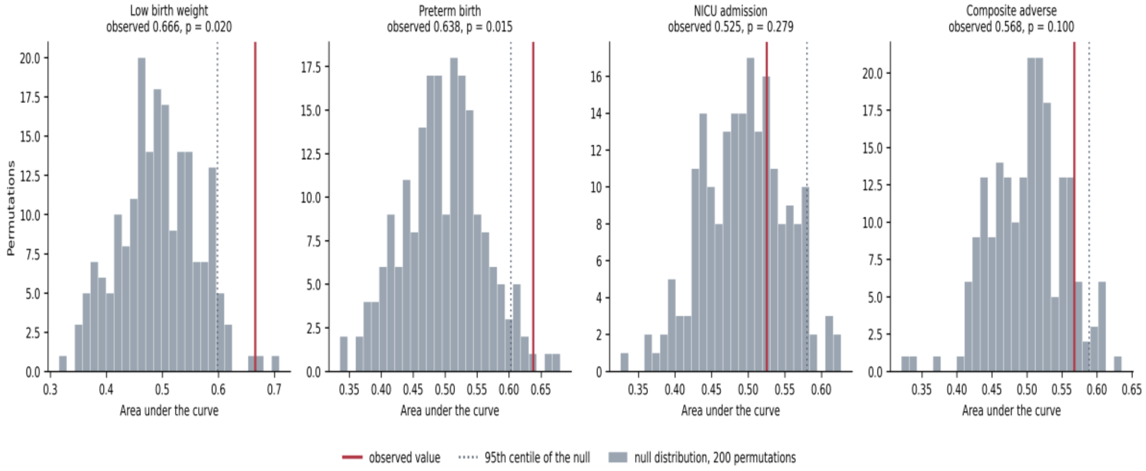

Figure S2. Null distributions obtained by permuting the outcome labels, with the observed value marked.
